# Comparison of ^18^F‐FDG PET/CT and ^68^Ga‐DOTATATE PET/CT in the Targeted Imaging of Culprit Tumors Causing Osteomalacia

**DOI:** 10.1111/os.12980

**Published:** 2021-03-11

**Authors:** Hao‐nan Yu, Ling Liu, Qiu‐song Chen, Qing He, Yan‐sheng Li, Ying Wang, Shuo Gao

**Affiliations:** ^1^ Department of PET‐CT Diagnostic Tianjin Medical University General Hospital Tianjin China; ^2^ Department of Traditional Chinese Medicine Tianjin 4th Center Hospital Tianjin China; ^3^ Department of Endocrinology and Metabolism Tianjin Medical University General Hospital Tianjin China

**Keywords:** ^18^F‐FDG, ^68^Ga‐DOTATATE, Hypophosphatemia, PET/CT, Tumor‐induced osteomalacia

## Abstract

**Objective:**

To assess and compare the performance of fluorine‐18‐labeled fluorodeoxyglucose positron emission tomography (^18^F‐FDG‐PET/ CT) and gallium‐68‐labeled tetraazacyclododecanetetraacetic acid‐DPhe1‐Tyr3‐octreotate (^68^Ga‐ DOTATATE) PET/CT in the targeted imaging of culprit tumors causing osteomalacia.

**Methods:**

This was a clinical retrospective analysis. We analyzed 13 patients (five men, eight women; mean age, 49 years; range, 19–55 years) with suspicion of tumor‐induced osteomalacia (TIO) between March 2017 and October 2019. All patients underwent two functional imaging methods to locate the culprittumors. Studies were performed on a PET/CT scanner. The injection doses of ^18^F‐ FDG and ^68^Ga‐DOTATATE were 0.5mCi/kg and approximately 5.0mCi, respectively. In the two scans, the whole body was captured from head to toe 45 to 60 min after intravenous tracer injection. ^68^Ga‐DOTATATE PET/CT and ^18^F‐FDG PET/CT imaging results locate culprit tumors according to the following criteria: (i) abnormal foci uptake concentration was observed locally, and the uptake level was higher than the background level of the right lobe of the liver; (ii) combined CT showed or did not have obvious abnormal density changes; and (iii) non‐specific ingestion lesions due to fracture, arthritis, necrosis of femoral head are excluded. Compared with the results of pathological examination and clinical follow‐up, the sensitivity, specificity and accuracy of ^68^Ga‐DOTATATE PET/CT imaging and ^18^F‐FDG PET/CT imaging for TIO were analyzed.

**Results:**

All patients had symptoms of osteomalacia and hypophosphatemia. The lag time (symptoms to PET diagnosis) ranged from 2 to 12 years. There were eight cases of TIO patients and five cases of non‐TIO patients confirmed by surgery, pathology and follow‐up. Among the eight TIO patients, there were six cases (75.0%) of PMTs, one case (12.5%) of giant cell tumor, one case (12.5%) of hemangiopericutoma. Most (*n* = 6, 75.0%) of the confirmed tumors in our patient population were in the lower extremities, followed by craniofacial regions (*n* = 1, 12.5%), and torso (*n* = 1, 12.5%), respectively. Among the five non‐TIO patients, there were two cases of Fanconi syndrome, one case of rickets, and two cases of sporadic osteomalacia hypophosphorus. The culprit tumors could be located either in the bone (*n* = 5, 62.5%) or the soft tissue (*n* = 3, 37.5%). ^18^F‐FDG PET/CT was able to localize the tumor in six (6/13, 46.1%) patients. ^68^Ga‐DOTATATE PET/CT detected tumor in 8 (83.3%) of 13 patients. The sensitivity of ^68^Ga‐DOTATATE PET/CT imaging and ^18^F‐FDG PET/CT imaging in the evaluation of TIO in our patient population were 100% (8/8) *vs* 75% (6/8). The specificity of the two different methods was 80% (4/5). The overall accuracy was 92.3% (12/13) *vs* 76.9% (10/13).

**Conclusions:**

^68^Ga‐DOTATATE PET/CT is very effective in assessing hypophosphatemia patients with TIO typical symptoms compared with ^18^F‐FDG. Therefore, in clinically suspected cases of hypophosphatemic osteomalacia, ^68^Ga‐DOTATATE PET/CT should be preferred as an imaging modality investigation to avoid delay in the treatment of this disease.

## Graphical abstract

A 59‐year‐old man had difficulty working with worsening diffuse bone pain, for more than 3 years. His serum phosphate level was 0.60 mmol/L. ^68^Ga DOTATATE PET/CT image (A, C) demonstrated an intense activity with maximum standardized uptake value (SUV_max_) of 17.2 (arrow) behind the right patella (C, white arrow), whereas ^18^F‐FDG PET/CT image (B, E) revealed no increased uptake in the soft tissue in the same lesion. The finding was suggestive of a causative tumor. After surgically removing a tumor 1.2 cm in its largest dimension from the right knee, the patient's symptoms promptly improved, and his postsurgical serum level returned to normal at 1.09 mmol/L. The results of the pathological examination confirmed the phosphaturic mesenchymal tumor (F).

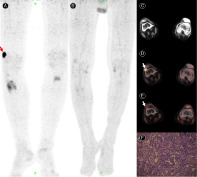

## Introduction

Tumor‐induced osteomalacia (TIO)—also known as oncogenic osteomalacia—is a rare paraneoplastic syndrome caused by the overproduction of fibroblast growth factor 23 (FGF23) by a tumor. TIO was first described by McCance in 1947^1^, and less than 500 cases have been reported in the literature so far. It is characterized by bone pain, muscular atrophy and pathologic fracture. Studies suggest that TIO affects men and women equally, and has an average onset age of 40–45 years but some cases have also been reported in children^2^. Biochemically, TIO is characterized by hypophosphataemia, phosphaturia, low serum active metabolite vitamin D (1,25 dihydroxyvitamin D_3_) levels, and usually increased levels of alkaline phosphatase^3^, ^4^. The disorders were mainly caused by benign FGF‐2‐producing mesenchymal tumor. FGF‐23 affected the reabsorption of phosphorus by the kidney, resulting in osteomalacia caused by increased renal phosphorus discharge. The majority of tumors are a distinct entity and are classified as phosphaturic mesenchymal tumors (PMTs) of the mixed connective tissue variant. Complete surgical resection leads to cure, however, the culprit tumors might be very difficult, or even impossible, to find as they can be anywhere in the body, in soft tissue or bone. In addition, Because of the slow‐growing nature of these benign tumors, the local symptoms directly imposed by the tumor are usually overshadowed by the severe systemic problems of osteomalacia, such as bone pain. Therefore, the key to cure this disease is to accurately locate the culprit tumor.

A stepwise approach to locating the PMT is advocated, functional imaging is the initial step, followed by anatomical imaging. Bone scanning, computerized tomography (CT), magnetic resonance imaging (MRI), Indium‐111 pentetreotide or octreotide scintigraphy, and positron emission tomography (PET) have all been employed in an effort to localize the tumor. PMTs are highly vascular tumors that demonstrate considerable tracer uptake. The most sensitive and specific functional imaging studies exploit the fact that PMTs express somatostatin receptors, mainly somatostatin receptor subtype 2A^5^. Then, they can be detected using SSTR‐PET/CT to locate the PMTs^6^. Recent literature data have demonstrated that SSTR‐PET/CT labeled with Gallium‐68 (^68^Ga), plays an important role in the detection and localization of culprit tumors in patients with TIO^7^. In this investigation, the efficacy of PET‐CT with a somatostatin‐receptor agent, ^68^Ga‐DOTATATE in the detection of tumors responsible for TIO was retrospectively assessed.^18^FDG‐PET/CT, which derives its utility from the greater metabolic activity of neoplastic tissue, is also a useful tool for locating PMTs, which are typically metabolically active. As these tumors are slow growing and commonly small in size, the FDG uptake may be variable. In some studies, ^18^F‐FDG PET/CT has shown good sensitivity for localizing TIO tumors in several case reports and studies^8^, ^9^. Although it is inferior to octreoscan in terms of its overall sensitivity, specificity, and positive and negative predictive values, tumors that were not seen on an octreoscan can at times be identified by ^18^FDG‐PET/CT imaging, and, therefore, the two types of imaging can be complemented.

However, evidence on the direct comparison of ^68^Ga‐DOTATATE PET/CT and ^18^F‐FDG PET/CT is limited. In this retrospective study, we scanned 13 patients suspected TIO to: (i) describe our experience with ^68^Ga‐DOTATATE PET/CT in 13 patients with TIO; (ii) assess and compare the efficacy of ^68^Ga‐DOTATATE PET/CT with ^18^F‐FDG PET/CT in detecting TIO; and (iii) find out the most suitable functional imaging method to target the culprit tumor.

## Subjects and Methods

### Inclusive and Exclusion Criteria

The clinical and imaging data of patients who met the following criteria in Tianjin Medical University General Hospital from March 2017 to October 2019 were analyzed.

Inclusion criteria were as follows: (i) clinical suspicion TIO; (ii) the patient underwent ^68^Ga‐DOTATATE PET/CT and ^18^F‐FDG PET/CT to locate the culprit tumor; (iii) the main evaluation index was the consistency of the diagnostic performances of ^68^Ga‐DOTATATE PET/CT and ^18^F‐FDG PET/CT with pathological and follow‐up results; and (iv) a retrospective study.

Exclusion criteria: (i) complicated with other malignant neoplastic diseases; (ii) age<18 years; and (iii) less than 12 months of follow‐up.

### Patient Information

Finally, 13 patients (five men, eight women; mean age, 49 years; range, 18–55 years) with suspicion of TIO was performed, all of whom were referred by the endocrinology department and all had severe bone pain and osteomalacia with hypophosphatemia. Biochemical evidence included decreased blood phosphorus, elevated urinary phosphorus, and normal or low blood calcium levels. After the lesions were found by PET/CT scan, enhanced computed tomography (CT) and magnetic resonance imaging (MRI) were performed to clarify the scope and size of the tumor and its relationship with adjacent tissues. The study was approved by the interdepartmental review committee. Informed written consent was also obtained from all the patients.

### Functional Imaging method

#### *^18^F‐FDG* PET/CT Imaging

Studies were performed on a PET/CT scanner (Discovery 710; GE Healthcare, Milwaukee, WI). All the patients fasted for at least 6 h before the ^18^F‐FDG injection, and blood glucose was ensured to be less than 150 mg/dL at the time of injection. Whole‐body acquisition from head to toe was started 45 to 60 min after IV injection of 3.7 MBq/kg of ^18^F‐FDG. A low‐dose, non‐contrast CT was used for attenuation correction and anatomic localization. Maximum standardized uptake values (SUV_max_) were normalized by body weight.

#### *^68^Ga‐DOTATATE* PET/CT Imaging

DOTATATE is an amide of the acid DOTA (1,4,7,10‐tetraazacyclododecane‐1,4,7,10‐tetraacetic acid), which acts as a chelator for the radionuclide to (Tyr3)‐octreotate, a derivative of octreotide. ^68^Ga‐DOTATATE PET/CT has been approved by the US Food and Drug Administration for clinical use. Through a peripheral vein, approximately 5.0mCi of ^68^Ga‐DOTATATE was administered. After approximately 45–60 min, the patient was positioned in the PET/CT scanner, and images from the toes to top of the skull.

### Image Interpretation

^68^Ga‐DOTATATE PET/CT and ^18^F‐FDG PET/CT Imaging results showed that suspected culprit tumor meeting the following criteria were positive: (i) abnormal foci uptake concentration was observed locally, and the uptake level was higher than the background level of the right lobe of the liver; (ii) combined CT showed or did not have obvious abnormal density changes; and (iii) non‐specific ingestion lesions due to fracture, arthritis, necrosis of femoral head are excluded. The ^68^Ga‐DOTATATE PET/CT and ^18^F‐FDG PET/CT images were interpreted by two experienced nuclear medicine physicians who were unaware of other clinical and imaging information.

### Observer Study

The main observer indicators were as follows: (i) clinical characteristic data: gender, age and clinical manifestations; (ii) tumor related conditions: distribution range, pathological types; (iii) functional imaging data: SUV_max_ of of lesions in two different functional imaging examinations; and (iv) the diagnostic efficacy of the two methods included sensitivity, specificity, positive predictive value, negative predictive value, and accuracy.

### Statistical Analysis

Statistical analysis was conducted using SPSS version 20.0 software. The diagnostic efficacy of ^68^Ga‐DOTATATE PET/CT imaging and ^18^F‐FDG PET/CT imaging for the culprit tumors causing osteomalacia was statistically compared with the results of surgical pathology and postoperative follow‐up.

## Result

### Patient's Characteristic and Biochemical Examination

Thirteen patients were included, with a mean follow‐up time of 12 months (range of 6 to 16 months). The lag time (symptoms to PET diagnosis) ranged from 2 to 12 years. All patients had symptoms of bone pain and hypophosphatemia. Symptoms of 13 patients included bone pain (13/13, 100%), muscle weakness (9/13, 69.2%), spine malformation (7/13, 53.8%), pathological fracture (2/14, 14.3%). Bone pain is the most common symptom and the initial symptom of most patients, most of which starts in the lower limbs and lower back. Two patients had pathological fractures located at the upper left fibula and lumbar spine. The clinical characteristics, biochemical examination and histopathologic findings are summarized in Table 1.

**TABLE 1:** Characteristics and histopathological findings of 13 patients suspected with tumor‐induced Osteomalacia

Initial characteristics	Date
Age (years)	49 (18–55)
Sex	
Male	5 (38.4%)
Female	8 (61.5%)
Symptom and signs	
Osteodynia	13 (100%)
Muscular atrophy	11 (84.6%)
Spine malformation	9 (69.2%)
Pathological fracture	2 (15.4%)
Biochemical Criterion	
Serum phosphate (mmol/L)	0.51 ± 0.16 (Normal reference, 0.8–1.45)
Serumjavascript:; calcium (mmol/L)	2.1 ± 0.2 (Normal reference, 2.15–2.55)
Urine phosphorus (mmol/L)	13.69 ± 5.28 (Normal reference, 4.65–21.12)
Final Diagnosis	
TIO	8 (61.5%)
Fanconi syndrome	2 (15.4%)
Sporadic osteomalacia hypophosphorus	2 (15.4%)
Rickets	1 (7.6%)
HistoPathological findings	
PMTs	6 (75%)
Giant cell tumor	1 (12.5%)
Hemangiopericutoma	1 (12.5%)

### Locations and Types of the Tumors

There were eight cases of TIO patients and 5 cases of non‐TIO patients confirmed by surgery, pathology and follow‐up.

Among the eight TIO patients, there were six cases (75.0%) of PMTs, one case (12.5%) of giant cell tumor, one case (12.5%) of hemangiopericutoma. The pathogenic tumors of the TIO patients were all single benign lesions. The symptoms of all of these eight patients were completely relieved postresection of the causative tumors. In addition, their serum phosphate levels returned to normal after the surgery.

Among the five non‐TIO patients, there were two cases of Fanconi syndrome, one case of rickets, and the other two cases had no clear etiology of hypophosphoric soft bone disease. After treatment with neutral phosphorus and calcitriol, the symptoms of the two patients with unknown causes were significantly relieved and the blood phosphorus remained normal, and they were diagnosed as sporadic osteomalacia hypophosphorus.

**Fig. 1:**
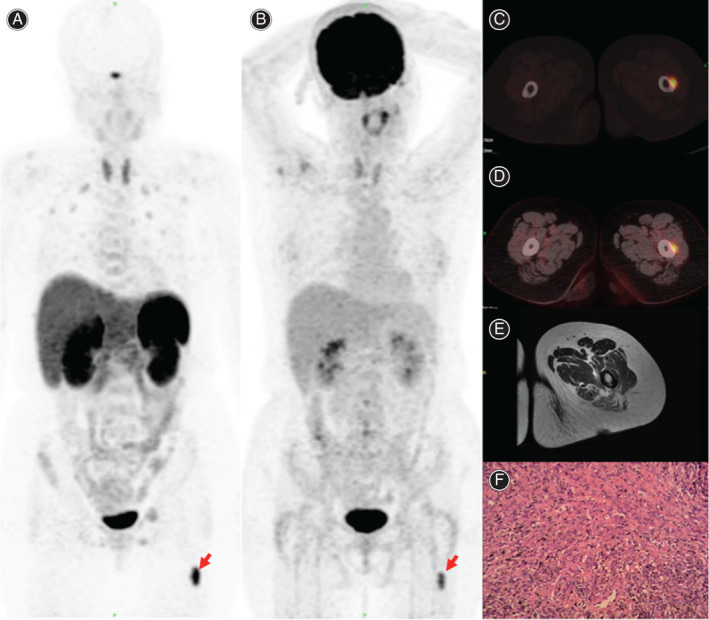
A 56‐year‐old female with osteoporosis for 3 years and Blood phosphorus 0.23 mmol/L underwent both entire body ^18^F‐FDG and ^68^Ga DOTATATE PET/CT studies from head to toe (lower limbs images are not shown here). ^18^F‐FDG PET/CT Maximum Intensity Projection (MIP) (A) and ^68^Ga‐ DOTATATE MIP image (B) showed intense tracer uptake in the left femur (red arrow). Additionally, ^18^F‐FDG PET/CT (C) and ^68^Ga DOTATATE PET/CT (D) transaxial fused images localize the abnormal uptake to an ill‐defined lesion in the left middle femur and bone destruction in the corresponding area. Subsequently guided MRI was performed, which showed heterogeneous signal in T2WI and restricted diffusion in DWI (E) corresponding to the PET abnormality. The lesion was excised and histopathology revealed phosphaturic mesenchymal tumor (F). Patient's symptoms improved dramatically after excision of the lesion.

The culprit tumors could be located either in the bones (*n* = 5, 62.5%) or the soft tissue (*n* = 3, 37.5%). Most (*n* = 6, 75.0%) of the confirmed tumors in our patient population were in the lower extremities, followed by craniofacial regions (*n* = 1, 12.5%), and torso (*n* = 1, 12.5%), respectively (Fig. 1).

### Functional Imaging Diagnostic Performance

Finally, culprit tumors were successfully located in eight patients and confirmed by histopathology. ^18^F‐FDG PET/CT was able to localize the tumor in 6 (6/13, 46.1%) patients. ^68^Ga‐DOTATATE PET/CT detected tumor in eight (83.3%) of 13 patients. Further CT and MRI examinations of the corresponding sites were performed on the focus with positive PET/CT, and the positive rate was 6/8 (75%) 8/8 (100%), respectively. The sensitivity of ^68^Ga‐DOTATATE PET/CT imaging and ^18^F‐FDG PET/CT imaging in the evaluation of TIO in our patient population are therefore 100% (8/8) *vs* 75% (6/8). The specificity of the two different methods was 80% (4/5). The overall accuracy is 92.3% (12/13) vs 76.9% (10/13). Diagnostic efficiency of different imaging methods showed in Table 2.

**TABLE 2:** Diagnostic efficacy of different imaging methods for tumor‐induced osteomalacia

Function Imaging	Number	Sensitivity	Specificity	PPV	NPV	Accuracy
^68^Ga‐DOTATATE PET/CT	13	100%	80%	88.9(8/9)	100%(4/4)	92.3% (12/13)
^18^F‐FDG PET/CT	13	75% (6/8)	80% (4/5)	85.7(6/7)	66.7%(4/6)	76.9% (10/13)

**Fig. 2:**
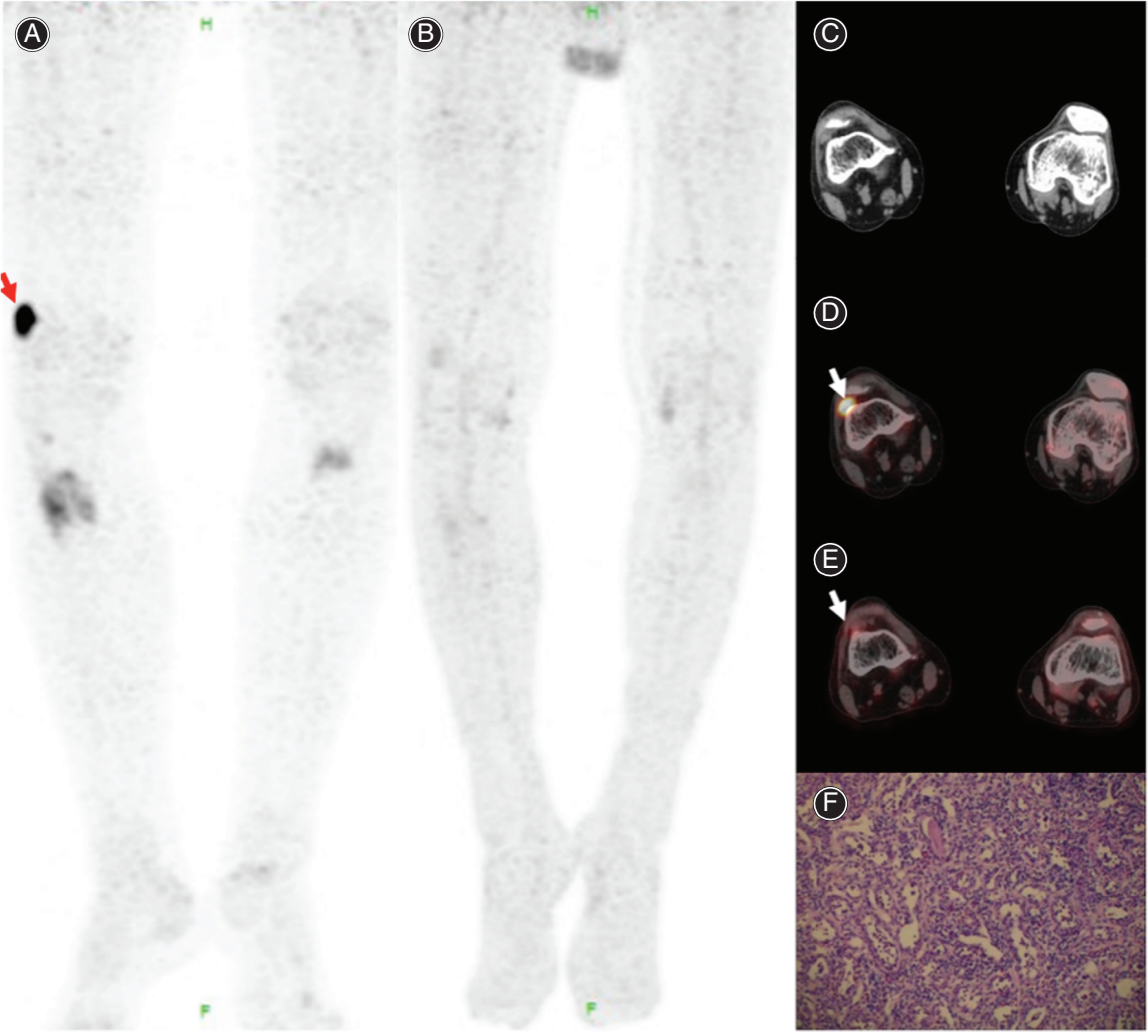
A 59‐year‐old man had difficulty working with worsening diffuse bone pain, for more than 3 years. His serum phosphate level was 0.60 mmol/L. ^68^Ga DOTATATE PET/CT image (A, C) demonstrated an intense activity with maximum standardized uptake value (SUV_max_) of 17.2 (arrow) behind the right patella (C, white arrow), whereas ^18^F‐FDG PET/CT image (B, E) revealed no increased uptake in the soft tissue in the same lesion. The finding was suggestive of a causative tumor. After surgically removing a tumor 1.2 cm in largest dimension from the right knee, the patient's symptoms promptly improved, and his postsurgical serum level returned to normal at 1.09 mmol/L. The results of the pathological examination confirmed the phosphaturic mesenchymal tumor (F).

In patient 3 (Fig. 2) and patient 8, ^68^Ga‐DOTATATE PET/CT showed high uptake foci in the soft tissue nodules behind right patella and right femur, respectively. However, ^18^F‐FDG PET/CT showed no significant increase in uptake in the same lesions. The lesion was excised and confirmed as giant cell tumor of tendon sheath and PMT on histopathology, respectively.

There is one true false‐positive case in a 19‐year‐old female. ^68^Ga‐DOTATATE PET/CT scan revealed activity in her right calcaneal bone. However, MR examination showed edema of bone marrow in the right calcaneal bone and low signal in T1WI and high signal in T2WI in the surrounding soft tissue, suggesting inflammatory lesions. Instead of surgical resection of the DOTATATE positive lesion, the patient received medical treatment, oral phosphorus, calcium and vitamin D supplementation, and followed‐up. The blood phosphorus level of the patient recovered, and the bone pain was relieved. The case was eventually diagnosed as hypophosphatemic rickets.

In four patients with negative ^68^Ga DOTATATE PET/CT scan, there was no false‐negative because these patients eventually either had diagnoses of Fanconi syndrome (*n* = 2) or sporadic osteomalacia hypophosphorus (*n* = 2). In the 1‐year follow‐up examination, all these five patients had normal serumphosphate level, which excluded a diagnosis of TIO.

**Fig. 3:**
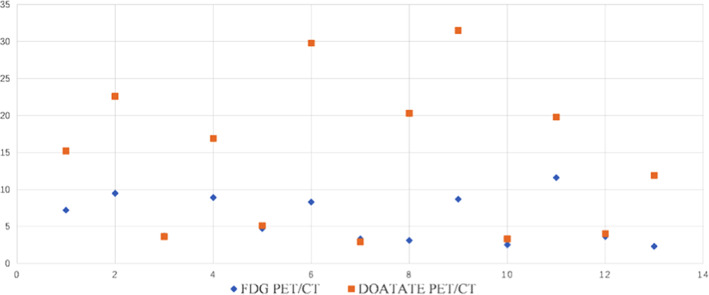
SUVmax of lesion in two different functional imaging examinations.

The lesion‐to‐background contrast was better in ^68^Ga‐DOTATATE PET/CT scan compared with that in ^18^F‐FDG PET/CT scan, enabling confident diagnosis. The absorption of ^68^Ga‐DOTATATE was stronger than that of ^18^F‐FDG, and the SUV_max_ was higher (Fig. 3).

## Discussion

TIO is a rare clinical manifestation of acquired low‐phosphorus osteomalacia. TIO is clinically significant but non‐specific. Symptoms mainly include progressive bone pain, limb weakness, limited mobility, and shortened height, which seriously affect the patient's quality of life. The disease is often misdiagnosed as ankylosing spondylitis, osteoporosis, lumbar intervertebral disc process^10^. When patients' biochemical examination results show that blood phosphorus level decreases, urine phosphorus excretion increases, phosphorus clearance index decreases, alkaline phosphatase increases, blood L, 25 (OH) and D_3_ levels decrease, TIO should be taken into account. However, osteomalacia due to hereditary hypophosphate, renal tubular acidosis, Fanconi syndrome, primary hyperparathyroidism and other causes should be excluded.

### Targeted Imaging of Culprit Tumors

Most of TIO's culprit tumors are benign and originate from mesenchymal tissue. The most common pathological type is phosphate urinary mesenchymal tumor. Other pathological types include giant cell tumor of tendon sheath, hemangiectoma, Hodgkin's lymphoma and polyosseous fibrodysplasia syndrome^11^, ^12^. The pathophysiological mechanism is that the level of fibroblast growth factor (FGF)23 secreted by the culprit tumor in the blood is increased, which inhibits the activity of 1α‐hydroxylase in the kidney and the expression of sodium/phosphorus co‐transporter, leading to increased urine phosphorus excretion and reduced absorption of phosphorus in the small intestine, resulting in hypophosphatemia^13^. PMTs are reported to express a variety of somatostatin receptors (SSTR1, 2A, 2B, 3, 4, 5). The use of somatostatin receptor‐based scans in TIO has been well described. Most previous reports using Indium‐111 labeled octreotides. Recently, ^99^mTc labeled octreotide whole body scan also showed utilization in the evaluation of TIO^14^. Breer *et al*. reported immunohistochemical staining on 15 PMTS from 14 patients with TIO, and found diffuse and strongly positive SSTR2A staining in all tumors^15^. Antunes *et al*. found that ^68^Ga‐DOTATATE had a stronger affinity for SSTR2 receptors than other Somatostatin receptor‐based scans and higher tumor uptake^16^. In addition, some studies compared the clinical value of various somatostatin receptor based scans in patients with neuroendocrine tumor and found that ^68^Ga‐DOTATATE PET/CT had more advantages in detecting small lesions and those with relatively complex anatomical structures. Therefore, ^68^Ga‐dotatate was selected in our study.

### Function Imaging for the TIO Detection

Patients have a unique opportunity of complete cure from their debilitating osteomalacia if these tumors can be localized and excised. However, localizing these often small sized, clinically inapparent tumors that can be present anywhere in the body from head to toe is challenging. Hence, functional imaging plays an important role in the management of TIO before proceeding to anatomical imaging. In this case series, we describe our experience with different functional imaging modalities in TIO.

#### TIO Detection with FDG PET/CT

^18^F‐FDG is the most widely used radiotracer in nuclear medicine and also the first PET tracer reported in literature to be used in the detection of TIO^17^. The utility of ^18^F‐FDG PET/CT in the localization of tumors in TIO has been described in several case reports and a few case series ^9^, ^18^. However, ^18^F‐FDG PET/CT lacks specificity, and false‐positive uptake in nonspecific inflammatory areas is common. The reported sensitivity of ^18^F‐FDG PET/CT of published studies in detecting TIO varied greatly from 36.3% to 88%, with the pooled sensitivity being 67%. In our current series of patients with TIO, ^18^F ‐FDG PET/CT was false negative in two out of 10 patients, thus suggesting limitations in its utility. The relatively poor performance of ^18^F‐FDG PET/CT might be mainly due to the benign nature and low metabolic activity of the mesenchymal tumors associated with TIO.

#### TIO Detection with SSTR PET/CT

The systematic review and meta‐analysis showed that since SSTRs is highly expressed in most of the culprit tumors that cause osteomalcic, the culprit tumors can be well diagnosed in the exploration of TIO patients using SSTRS‐PET/CT. Even if false negative findings are possible, in about 10% of cases, it should be underlined that SSTR‐PET/CT has allowed the detection of culprit tumors which remained occult with conventional imaging methods in most of the cases^19^, ^20^. The main reason for the false negative results of conventional imaging methods is that the primary culprit of osteomalacia is the small size and variable location of the tumor^21^. The ^68^Ga‐DOTATATE PET/CT results of Patient 8 showed positive uptake in the left calcaneus, but both MR and bone tissue puncture examination suggested inflammation, which was confirmed by follow‐up. The false positive of SSTR‐PET/CT is due to inflammatory lesions caused by overexpression of SSTR by activated inflammatory cells^22^, ^23^. Granulomatous lesions may be positive at SSTR‐PET/CT in some cases and it could be difficult to differentiate them from tumors causing osteomalacia—or neuroendocrine tumors—using conventional imaging methods or SSTR‐PET/CT. MRI is of great value in accurately showing tumor boundary and range in the diagnosis of bone and soft tissue tumors, and differentiating TIO pathogenic tumor and non‐specific uptake caused by fracture inflammatory response in PET/CT imaging.

### Diversity of Lesion Distribution

Approximately 53% of PMTs occur in bone, 45% in soft tissue, and 3% in the skin. Most arise within the extremities, whereas occurrence within the head and neck has been described in only 5% of cases, with the paranasal sinuses being the most favored sites in this region. Involvement of the mandible and mandibular soft tissues is exceptionally rare^24^. In the present study, (5/8) 67.5% of tumor was localized within the bone, (3/8) 37.5% in the soft tissue and (7/8) 87.5% in the lower limb. Because of their small size, slow‐growing nature, and location in atypical sites, detection of these tumors is often challenging. Thus, definitive treatment is often delayed by an average of 5 years from diagnosis due to difficulty in tumor localization^25^.

Some helpful observations of this study need to be emphasized again. First, focus should be placed on making sure that the PET/CT imaging covers the entire body, from head to toe, including the hands and feet. Second, be alert to the possibility of false positive inflammation, as shown in Patient 5. In patients with TIO, any abnormal focally intense ^68^Ga‐DOTATATE uptake is suggestive of a tumor. If the uptake level of the lesion is not strong, MR examination should be performed to improve diagnostic accuracy. Third, other causes of hypophosphatemia, such as Fanconi syndrome, should be fully excluded before examination.

### Conclusion

Our results show that ^68^Ga‐DOTATATE PET/CT is very effective in assessing hypophosphatemia patients with TIO typical symptoms compared with ^18^F‐FDG. ^68^Ga‐DOTATATE PET/CT is a useful imaging modality in the targeted imaging of culprit tumors causing osteomalacia.
